# Editorial: Pain in Early and Late-Life: Capturing the Understudied Tails of the Lifespan Spectrum

**DOI:** 10.3389/fnins.2022.905479

**Published:** 2022-07-18

**Authors:** Jonas Tesarz, Marina López-Solà, Marta Čeko, Pavel Goldstein

**Affiliations:** ^1^Department of General Internal Medicine and Psychosomatics, University Hospital Heidelberg, Heidelberg University, Heidelberg, Germany; ^2^Serra Hunter Program, Unit of Psychological Medicine, Department of Medicine, School of Medicine and Health Sciences, University of Barcelona, Barcelona, Spain; ^3^Institute of Cognitive Science, University of Colorado, Boulder, CO, United States; ^4^The School of Public Health, University of Haifa, Haifa, Israel

**Keywords:** children, newborn, frailty, lifespan, chronic pain, elderly

Chronic pain remains a leading cause of long-term disability (GBD 2016 Disease Injury Incidence Prevalence Collaborators, 2017), affects 20% of the world's population, imposes higher costs on the society than heart disease, cancer, and diabetes combined (GBD 2016 Disease Injury Incidence Prevalence Collaborators, 2017; Buchbinder et al., 2018), and is associated with increased mortality (Tesarz et al., 2019). Despite this global relevance, therapeutic options remain unsatisfactory to date (Williams et al., 2020). Consequently, in recent years, enormous efforts have been made to expand the knowledge about the development and maintenance of chronic pain disorders and to improve treatment options. The dramatic nature of the situation and the increasing global health burden due to chronic pain even culminated recently in a “global call for action” (Buchbinder et al., 2018).

This resulted in a large body of research that has led to groundbreaking insights into the complex biopsychosocial mechanisms of pain processing and pain chronification. These efforts have substantially advanced knowledge of the mechanisms underlying pain, most recently leading to underlying changes in definition (Raja et al., 2020), the development of the concept of nociplastic pain (Fitzcharles et al., 2021), and the adoption of the ICD-diagnostic classification (Treede et al., 2019) of chronic pain. However, despite these far-reaching developments, treatment effects have stagnated and large portions of patient populations continue to face unsatisfactory treatment outcomes. This is especially true for vulnerable patient populations and specific marginalized groups in the population. The very young and the very old are also particularly affected by this “gap in knowledge about understanding and caring for” chronic pain conditions. The research has typically focused on adults with chronic pain conditions, often neglecting to examine specific age groups that are particularly vulnerable to chronic pain or face greater challenges in managing it, such as adolescents and the elderly. Thus, little is known about the specific mechanisms and characteristics of chronic pain in these age groups, which is critical for developing the most effective treatment strategies.

The goal of this special-topic issue of Frontiers is to bring together relevant research studies in the field of pain processing and neural mechanisms of pain across the tails of the lifespan spectrum: Pain in adolescent and geriatric patients.

We are using a series of articles to shed more light on this complex topic area. First, a topical review addresses common pediatric chronic head and facial pain syndromes and recommended treatment approaches (Sangalli et al.). It is not only the clinical relevance of these pain conditions in children and adolescents that makes this article so noteworthy. Rather, the presented findings make it clear that pain disorders in children represent a separate entity phenomenologically as well as epidemiologically and clinically, and that findings from adults cannot simply be extrapolated to this patient group. At the same time, the treatment of pediatric pain syndromes always requires a systemic view of the environmental factors and the involvement of caregivers. In the study by O'Sullivan et al., the authors exemplify the possibility of using ecological momentary assessments (EMA) to gain insight into the nature of children's “everyday” pain experiences in the family home environment. Digital daily reporting with the focus on “everyday” events is becoming increasingly important for exploring a child's pain fluctuations without disrupting the home environment. The study by Wiwe Lipsker et al. supports this perspective, showing the interaction between neuropsychological development and chronic pain, and demonstrating that chronic pain profoundly influences further development in children. Finally, particular pain syndromes (Payano Sosa et al.) and pain trajectories (Wettstein et al.) are investigated in old and very old people. Specifically, Payano Sosa et al. investigated somatosensory profiles in Burning Mouth Syndrome (BMS) Type 1 to highlight the cyclical effect of BMS pathophysiology across the day, boosting a personalized treatment fit for BMS patients. Little is known to date about the longitudinal trajectories of pain in old age and at the end of life. Wettstein et al. address the role of psychosocial factors such as eudaimonic wellbeing or personality as potential determinants of pain trajectories in late life.

This series of articles is intended to examine the understudied ends of the lifespan spectrum, and also to represent a “Call for Action” to target future research efforts in these unrepresented and critical areas in particular.

## Author Contributions

All authors listed have made a substantial, direct, and intellectual contribution to the work and approved it for publication.

## Conflict of Interest

The authors declare that the research was conducted in the absence of any commercial or financial relationships that could be construed as a potential conflict of interest.

## Publisher's Note

All claims expressed in this article are solely those of the authors and do not necessarily represent those of their affiliated organizations, or those of the publisher, the editors and the reviewers. Any product that may be evaluated in this article, or claim that may be made by its manufacturer, is not guaranteed or endorsed by the publisher.
